# Severe Fever with Thrombocytopenia Syndrome Virus among Domesticated Animals, China

**DOI:** 10.3201/eid1905.120245

**Published:** 2013-05

**Authors:** Guoyu Niu, Jiandong Li, Mifang Liang, Xiaolin Jiang, Mei Jiang, Haiying Yin, Zhidian Wang, Chuan Li, Quanfu Zhang, Cong Jin, Xianjun Wang, Shujun Ding, Zheng Xing, Shiwen Wang, Zhenqiang Bi, Dexin Li

**Affiliations:** Key Laboratory for Medical Virology–Chinese Center for Disease Control and Prevention, Beijing, People’s Republic of China (G. Niu, J. Li, M. Liang, C. Li, Q. Zhang, C. Jin, S. Wang, D. Li);; Shandong Province Center for Disease Control and Prevention, Jinan, China (X. Jiang, X. Wang, S. Ding, Z. Bi);; Yantai City Center for Disease Control and Prevention, Yantai, China (M. Jiang);; Laizhou County Center for Disease Control and Prevention, Laizhou, China (H. Yin);; Penglai County Center for Disease Control and Prevention, Penglai, China (Z. Wang);; Nanjing University Medical School, Nanjing, China (Z. Xing)

**Keywords:** severe fever with thrombocytopenia syndrome virus, SFTSV, infection, domesticated animals, host, China, Phlebovirus, family Bunyaviridae, pathogenic disease, transmission, viruses, zoonoses, prevalence

## Abstract

To investigate the infections of severe fever with thrombocytopenia syndrome virus (SFTSV) in domesticated animals, we sampled a total of 3,039 animals in 2 counties in Shandong Province, People’s Republic of China, from April to November 2011. SFTSV-specific antibodies were detected in 328 (69.5%) of 472 sheep, 509 (60.5%) of 842 cattle, 136 (37.9%) of 359 dogs, 26 (3.1%) of 839 pigs, and 250 (47.4%) of 527 chickens. SFTSV RNA was detected in all sampled animal species, but the prevalence was low, ranging from 1.7% to 5.3%. A cohort study in 38 sheep was conducted to determine when seroconversion to SFTSV occured. SFTSVs were isolated from sheep, cattle, and dogs and shared >95% sequence homology with human isolates from the same disease-endemic regions. These findings demonstrate that natural infections of SFTSV occur in several domesticated animal hosts in disease-endemic areas and that the virus has a wide host range.

Severe fever with thrombocytopenia syndrome virus (SFTSV) is a newly identified pathogenic member of the *Phlebovirus* species in the family *Bunyaviridae*, which in humans causes fever with thrombocytopenia syndrome (SFTS) (*1*). The common signs and symptoms of SFTS include high fever, gastrointestinal symptoms, thrombocytopenia, leukocytopenia, and multiorgan dysfunction with an average case-fatality rate of 10%–16%, according to the information system for disease control and prevention, Chinese Center for Disease Control and Prevention (China CDC). In severe SFTS cases, neural symptoms, hemorrhages, disseminated intravascular coagulation, and multiorgan failure can occur and may result in death (*2*).

SFTS has been reported in at least 13 provinces in the central, eastern, and northeastern regions of the People’s Republic of China. Most patients are farmers living in wooded, hilly, or mountainous areas, and the epidemic season is from March through November, with the peak incidence usually in June and July (*1*). Although *Haemophysalis longicornis* ticks have been implicated as vectors of SFTSV (*1**,**3*), and high seroprevalence to SFTSV has been reported in goats *(**4**,**5*), the host range of the virus has not been determined. The role of domesticated animals in the circulation and transmission of SFTSV remains unclear.

To assess the prevalence of SFTSV infections in domesticated animals, combined cross-sectional and cohort studies were conducted in Laizhou and Penglai in Shandong Province. This study area was selected on the basis of its high incidence of human SFTS cases in 2010 (Technical Appendix Figure 1). We report the detection of viral RNA and antibodies in sheep, cattle, dogs, pigs, and chickens. Our findings may provide valuable insights for understanding SFTSV ecology and transmission among animals and from animals to humans.

## Materials and Methods

### Study Design and Sample Collection

Animal sampling took place in Laizhou (119°33′–120°18′E, 36°59′–37°28′N) and Penglai (120°35′–121°09′E, 37°25′–37°50′N), Shandong Province, China. The most common domesticated animal species in the region include sheep, cattle, dogs, pigs, and chickens (Technical Appendix). Animals of these species were sampled monthly in the villages where SFTS human cases were reported in 2010. Fifty to 100 animals of each species were sampled each month from April through November of 2011 and the serum samples were collected for detection of viral RNA, antibodies, and virus isolation. Goats were not surveyed because their populations are small in the sampling area.

Each sampled animal was marked with a unique label except for the pigs, which were sampled at the time of slaughter. Sampled animals were excluded in subsequent sampling events. In addition, to monitor new infections of SFTSV, a cohort of 38 sheep (Laizhou, n = 17; Penglai, n = 21) negative for SFTSV viral RNA and antibodies was established, and the animals were sampled every 10 days from June 20 through November 30. Serum samples were aliquoted and kept in cryovials in liquid nitrogen. Viral RNA and antibodies were first screened at the local CDC laboratories in Laizhou and Penglai, respectively, and the results were confirmed later in the national laboratory of China CDC. During the sampling period, serum samples from new SFTS patients in the study region in 2011 were also collected for virus isolation and genetic analysis.

### ELISA

Antibodies in sheep, cattle, dogs, pigs, and chickens against SFTSV were detected by using a double-antigen sandwich ELISA described previously (*6*). In brief, a His-tagged affinity chromatography purified recombinant nucleocapsid (N) protein of SFTSV (strain HB29) expressed in *Escherichia coli* was used as the coating antigen for 96-well plates, and horseradish peroxidase (HRP)–conjugated antigen was used for detection. Serum samples obtained from 46 sheep, 55 cattle, 30 dogs, 55 pigs, and 50 chickens originating from either Beijing or Hebei provinces, where no human SFTS cases had been reported, were used as negative controls, and convalescent-phase SFTS patient serum samples were used as positive controls. All serum samples including negative controls were tested at a dilution of 1:10. After 96-well plates were coated with purified N protein (0.2 μg/well), serum samples were added, followed by HRP-conjugated N protein. TMB peroxidase substrate (3,3′,5,5′tetramethylbenzidine and hydrogen peroxide [H_2_O_2_]) was used for color development, and substrate conversion was measured by using a DTX 880 multimode detector (Beckman Coulter, Brea, CA, USA) with an incidence wavelength of 450 nm and a reference wavelength of 620 nm.

Each sample was tested in triplicate within the same test run and mean optical density (OD) values for each sample were converted to a percentage of the positive control (PP) values by using the following equation: (mean of net OD of test sample/mean of net OD of positive control) × 100. The PP value frequency distribution resulting from analyses of serum samples from control animals was determined to be normal by using the χ^2^ goodness-of-fit test. Cutoff values were determined by adding 3 × SD to the mean of the PP values obtained from analyses of negative control serum samples. The resulted cutoff values were 15.6, 15.3, 14.4, 13.7, and 14.2 for sheep, cattle, dog, pig, and chicken serum samples, respectively. A sample was considered positive if the PP value was above the cutoff threshold. For the detection of antibodies in a dog that was found positive with viral RNA in the blood, an indirect IgG ELISA was used. Briefly, the plate was coated with purified N protein as described, 2-fold dilutions of the serum samples were added, followed by HRP-conjugated anti-dog IgG (Sigma, Saint Louis, MO, USA). Cutoff values for the assay were determined by adding 3 × SD to the mean of OD values resulting from analyses of negative control serum samples from non-infected dogs. Endpoint titers were expressed as the reciprocal of the highest dilution of the serum samples.

### Neutralization Assay

A microneutralization assay was performed to detect neutralizing antibodies against SFTSV as described (*1*). Serial dilutions of sheep serum samples collected at various time points were mixed with an equal volume of 100 50% tissue culture infective doses of SFTSV (strain HB29), and the mixture was incubated at 37°C for 1.5 h. The mixture was then added to cultured Vero cells in a 96-well plate in quadruplicate. The plates were incubated at 37°C with 5% CO_2_ for 7 days. Viral infection was assessed by an immunofluorescence assay with mouse polyclonal antibodies against SFTSV. End-point titers were expressed as the reciprocal of the highest dilution of the serum that prevented infection.

### TaqMan Quantitative Real-Time Reverse Transcription–PCR

A TaqMan quantitative real-time reverse transcription PCR (qRT-PCR) was performed on all animal serum samples by using a certified real-time RT-PCR kit for clinical diagnosis (SFDA registration no. 340166, China). This kit targets the small segment of SFTSV, with 98.6% sensitivity and 99.1% specificity as described (*7*). Viral RNA copy numbers were determined from amplification of a standard curve of positive control RNA, and the cutoff cycle threshold (Ct) value for a positive reaction was set at 35 cycles. The detection limit for this kit is 50 RNA copies (5 μL/test) or 10 copies/μL. A result was considered positive if a sample had a Ct value below the cutoff.

### Virus Isolation and Sequence Analysis

Serum samples collected from animals or SFTS patients in acute phase, newly occurred in the study region in 2011, were inoculated onto monolayers of Vero cells for virus isolation as described (*1*). Cells were cultured at 37°C in an incubator supplied with 5% CO_2_ with media changes done twice per week. The isolated virus was sequenced and subjected to phylogenetic analysis by using the neighbor-joining method with MEGA version 5 (*8*), and compared with previous published viral sequences of SFTSV strains isolated from 11 SFTS patients and *H. longicornis* ticks in 2010 (*1**,**3*).

### Statistical Analyses

The rates of SFTSV RNA and antibody detection in Penglai and Laizhou were calculated monthly by animal species, and the statistical significance was analyzed by using either the Pearson χ^2^ or continuity correction χ^2^ test. Analyses were performed by using the SPSS software version 16.0 (IBM, Armonk, NY, USA). Viral RNA copies among animal species were analyzed by using the Kruskal–Wallis test, performed by using GraphPad Prism software (ver. 5.0; GraphPad Software, Inc., San Diego, CA, USA). Values of p<0.05 were considered statistically significant.

### Ethical Considerations

According to the medical research regulation of the ministry of health, all studies involved in human samples were reviewed and approved by the ethics committee of China CDC, which uses international guidelines to ensure confidentiality, anonymity, and informed consent. Written informed consent was provided by the patients. The protocols for animal sampling have been approved by the animal care committee of China CDC.

## Results

### SFTSV Infection in Domesticated Animals by Month

**Figure 1:**
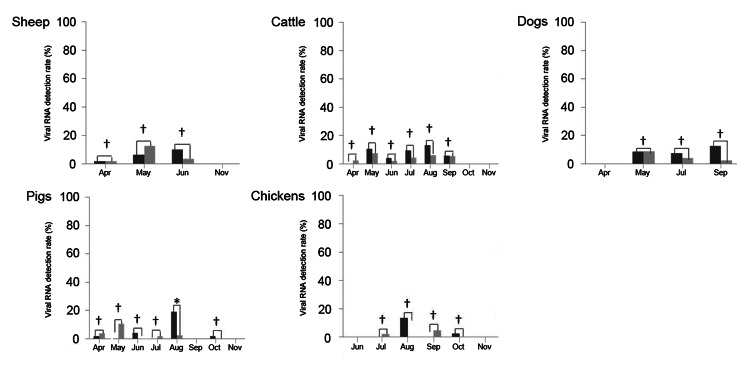
Serum severe fever with thrombocytopenia syndrome virus RNA detection rate in domestic animals from Laizhou and Penglai counties, China, April–November 2011. Viral RNA copies were detected by real-time reverse transcription PCR in serum samples from sheep, cattle, dogs, pigs, and chickens collected from Laizhou and Penglai counties in different months. The viral RNA detection rates are shown. Black bars indicate samples from Laizhou; gray bars indicate samples from Penglai. The viral RNA detection rate in 2 counties was compared (*p<0.05, †p>0.05).

We sampled 472 sheep, 842 cattle, 359 dogs, 839 pigs, and 527 chickens in Laizhou and Penglai counties to assess the prevalence of SFTSV RNA and antibodies. Our results showed that 3.8% (18/472) of sheep, 4.2% (35/842) of cattle, 5.3% (19/359) of dogs, 2.6% (22/839) of pigs, and 1.7% (9/527) of chickens were viral RNA-positive (Table). The monthly positive rates of viral RNA in sampled animals varied from April to November, ranging from 2% (n = 51) to 12.7% (n = 55) in sheep, 2.1% (n = 51) to 13.2% (n = 53) in cattle, 0 (n = 53) to 12.5% (n = 40) in dogs, 0 (n = 51) to 19.1% (n = 47) in pigs, and 0 (n = 40) to 13.5% (n = 37) in chickens, in which n is referred as to denominator of all animals sampled in that month (Figure 1). Although the overall positive rates for SFTSV varied between the 2 counties, the variance was not statistically significant (p>0.05). The median RNA concentration was 2.0 × 10^4^ copies/mL (95% CI: 1.6–2.6 × 10^4^) in sheep; 2.0 × 10^4^ copies/mL (95% CI: 1.5–2.9 × 10^4^) in cattle; 2.1 × 10^4^ copies/mL (95% CI: 0.96–4.7 × 10^4^) in dogs; 1.7 × 10^4^ copies/mL (95% CI: 1.4–2.1 × 10^4^) in pigs; and 1.7 × 10^4^ copies/mL (95% CI: 1.0–2.7 × 10^4^) in chickens (Technical Appendix Figure 2), and the differences among the studied animal species were not statistically significant (p = 0.87) (Technical Appendix Figure 2).

**Table:** Summary of SFTS virus viral RNA and specific antibodies detected in domesticated animals, China, 2011

Animal hosts	Laizhou Province				Penglai Province				Total		
	No. (%) RNA positive†	No. (%) antibody positive	No. serum samples tested		No. (%) RNA positive†	No. (%) antibody positive)	No. serum samples tested		No. (%) RNA positive†	No. (%) antibody positive	No. serum samples tested
Sheep	7 (3.4)	142 (69.3)	205		11 (4.1)	186 (69.7)	267		18 (3.8)	315 (69.5)	472
Cattle	22 (5.1)	233 (53.7)	433		13 (3.2)	276 (67.5)	409		35 (4.2)	442 (60.4)	842
Dogs	12 (6.6)	70 (38.5)	182		7 (4)	66 (37.3)	177		19 (5.3)	136 (37.9)	359
Pigs	13 (3.1)	3 (0.7)	426		9 (2.2)	23 (5.6)	413		22 (2.6)	26 (3.1)	839
Chickens	7 (2.7)	149 (57.1)	261		2 (0.8)	101 (38)	266		9 (1.7)	250 (47.4)	527

*SFTS, severe fever with thrombocytopenia syndrome virus. †Serum from sampled animals of each species was collected from April to November 2011.

**Figure 2:**
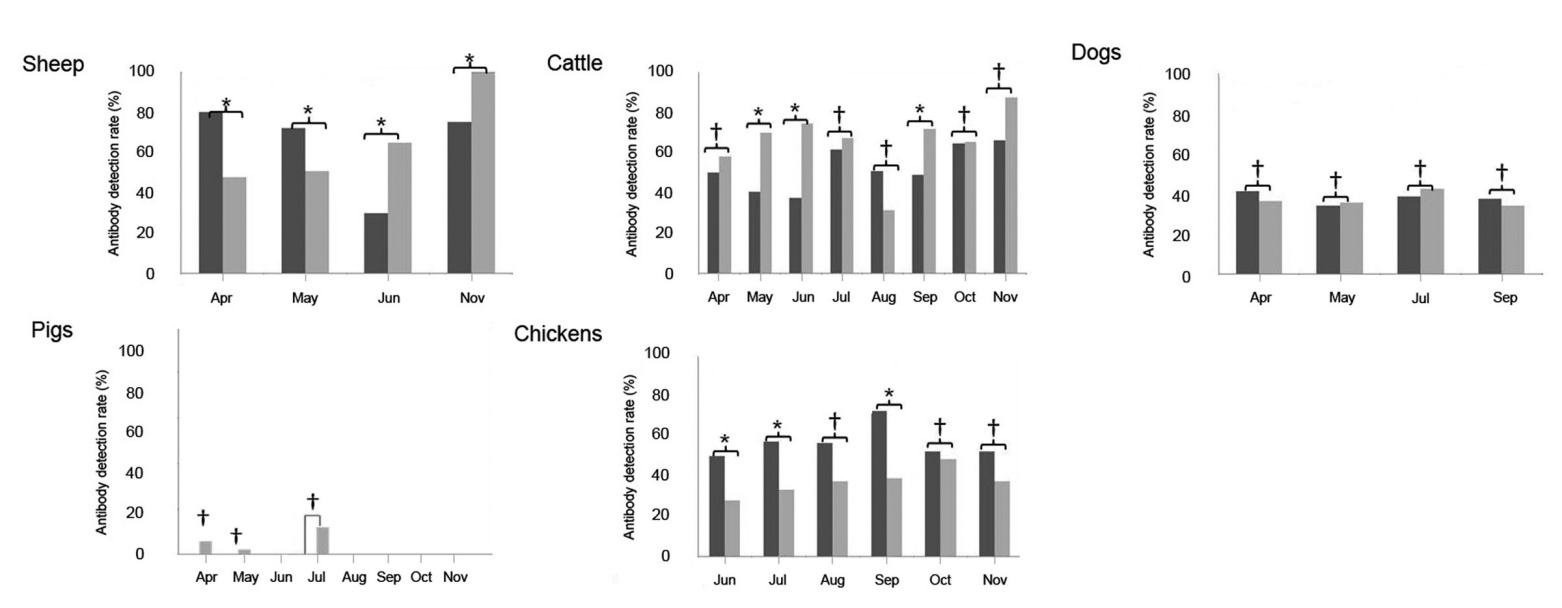
Serum antibody detection rate in domestic animals from Laizhou and Penglai counties, China, April–November 2011. Severe fever with thrombocytopenia syndrome virus N protein-specific antibodies were detected by double-antigen sandwich ELISA in serum samples of sheep, cattle, dogs, pigs, and chickens collected from Laizhou and Penglai counties in different months, and the antibody detection rates are presented. Black bars indicate samples from Laizhou; gray bars indicate samples from Penglai. The antibody detection rates in the 2 counties were compared (*p<0.05, †p>0.05).

Viral antibody prevalence was significantly higher (p<0.005) in sheep (69.5%, 328/472), cattle (60.4%, 509/ 842), dogs (37.9%, 136/359), and chickens (47.4%, 250/527) than in pigs (3.1%, 26/839) (Table; Technical Appendix Figure 3). As shown in Figure 2, monthly positive rates of antibodies in sheep varied from 30% (n = 30) to 80.4% (n = 51) in Laizhou County, and 48% (n = 50) to 100% (n = 82) in Penglai County during the sampling period; monthly positive rates in cattle varied from 37.5.3% (n = 48) to 66.3% (n = 80) in Laizhou, and from 31.3% (n = 48) to 78.7% (n = 87) in Penglai. In dogs, monthly positive rates ranged from 34.2% (n = 41) to 42.6% (n = 47) in the 2 regions, and the variation between sampling months was not statistically significant. Seroprevalence in pigs was low, and SFTSV antibodies were not detected in pig serum samples collected in August and September (n = 181). Monthly seroprevalence ranged from 28% (n = 25) to 72.7% (n = 44) in chickens in the regions.

### SFTSV Infection in a Cohort of 38 Sheep

**Figure 3:**
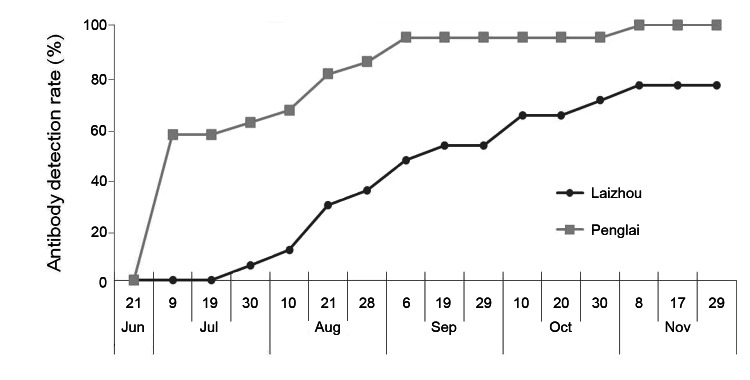
Detection of serum severe fever with thrombocytopenia syndrome virus (SFTSV) RNA and antibodies in a cohort of 38 sheep, China. Serum samples in a cohort of 38 sheep were collected from Laizhou and Penglai on the dates indicated on the x-axis from June 21 through November 29, 2011. SFTSV N protein–specific antibodies were measured by using a double-antigen sandwich ELISA, and the cumulative positive percentage in Laizhou and Penglai counties is presented along the timeline.

**Figure 4:**
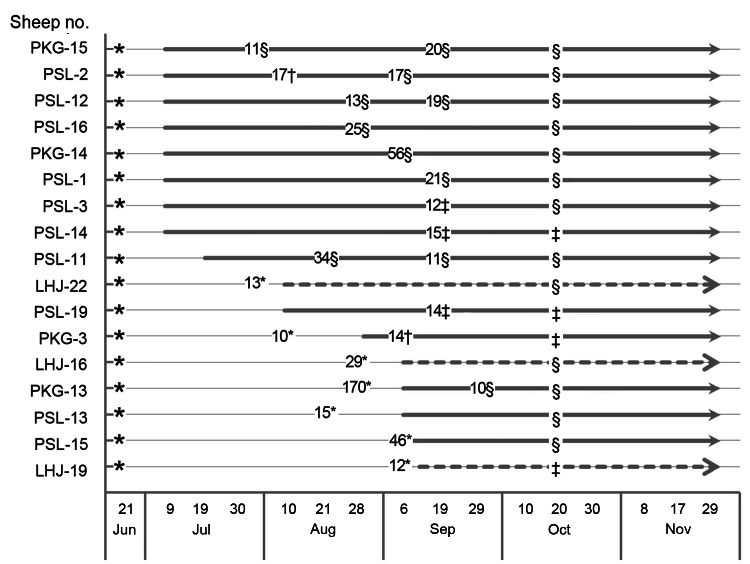
Time course of serum viral RNA and neutralizing antibodies in 17 sheep positive for severe fever with thrombocytopenia syndrome virus RNA, China. Detection of virus specific antibodies in Laizhou and Penglai is indicated by the dashed line and the bold line, respectively. The numbers on the lines indicate viral RNA copies (×10^3^/mL) in serum samples detected on the indicated dates. Neutralizing antibody levels in the initial samples collected on June 21, 2011, in the samples positive for viral RNA, and in the samples collected at the late stage on October 20, were measured by using a microneutralization assay. Neutralizing antibody titers are shown with different symbols: *neutralizing antibodies not detected; †neutralizing antibody titers = 4; ‡neutralizing antibody titers = 16; §neutralizing antibody titers >64.

In addition to the 472 sheep described above, a cohort of 38 sheep was monitored for SFTSV seroconversion during a 6-month period. As shown in Figure 3, seroconversion occurred in the cohort during the surveillance period, with an increasing seroprevalence that peaked at 76.5% (n = 17) in Laizhou and 100% (n = 21) in Penglai in November. In this cohort, 17 sheep were viral RNA positive either once or twice (6/17) over the sampling period. Viral RNA was mainly detected in August and September, and serum virus RNA quantities ranged from 10^4^ copies/mL to 1.7 × 10^5^ copies/mL (Sheep PKG-13). In 5 sheep, viral RNA appeared before seoconversion; but in 2 sheep (PSL-15, LHJ-19), viral RNA and SFTSV antibodies were detected on the same sampling day. In 10 of 17 sheep, viral RNA was detected once or twice after seroconversion (Figure 4).

Neutralizing antibody levels were also measured in 17 sheep serum samples on day 1 (June 21) and on the day when viral RNA was first detected, and again at a later stage (October 20). All tested samples were negative for neutralizing antibodies on day 1 but were positive at later times (Figure 4). No visible clinical signs of infection were detected in the cohort of sheep over the testing period regardless of antigen or antibody status.

### Course of SFTS Virus Infection in a Dog

**Figure 5:**
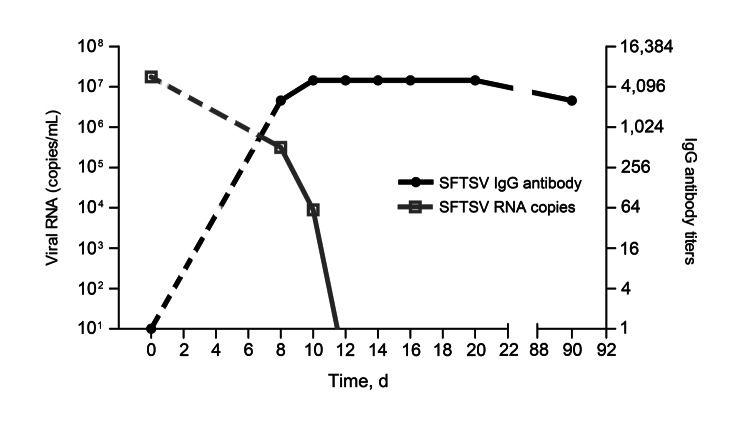
Time course of serum severe fever with thrombocytopenia syndrome virus (SFTSV) RNA and antibody in a naturally infected dog, China, 2011. SFTSV RNA copies and virus-specific antibodies were detected in serum samples from a dog on day 0, once every sample period of 2 days from day 8 to day 22, and on day 90. The gray open squares indicate viral copies; black circles indicate virus-specific IgG. The dashed lines indicate predicted time course of viral RNA and antibodies due to lack of data during day 1–day 7.

During the testing, we found 1 dog with a high viral RNA load (1.7 × 10^7^ copies/mL) and no antibodies. The dog was isolated, examined, and sampled daily from day 8 through day 22 and a sampled once more on day 90 (Figure 5). This dog had detectable viral RNA on days 8 and 10; levels declined thereafter, and levels were undetectable levels on day 12. Concurrently, SFTSV IgG titers reached 1:2,560 on day 10 and remained at that level through day 90. No signs of illness were observed during the entire period of isolation.

### Virus Isolation and Sequence Analysis

**Figure 6:**
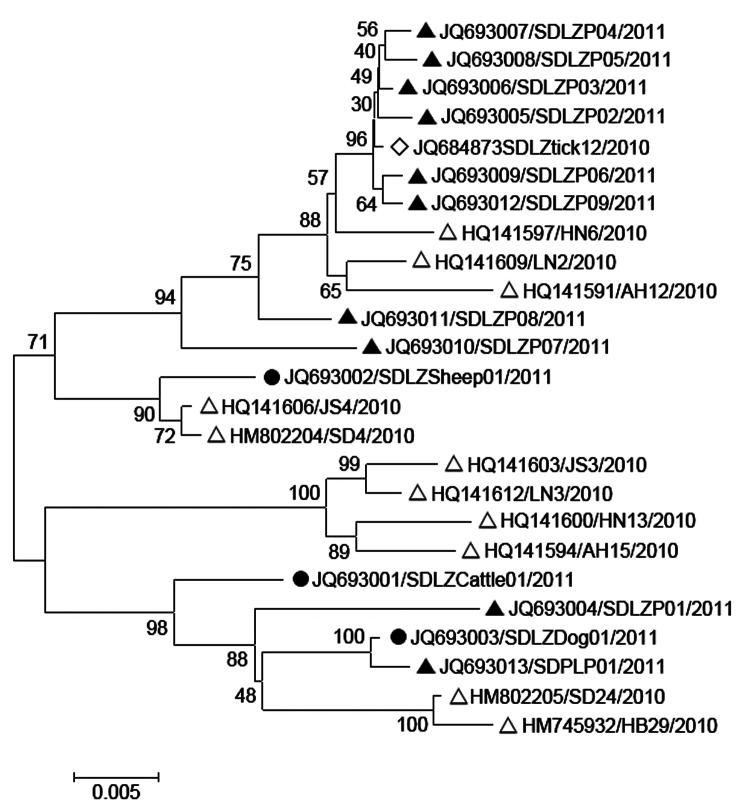
Phylogenetic analysis of severe fever with thrombocytopenia syndrome virus (SFTSV) isolates from domesticated animals. The evolutionary relationship of small segments of SFTSV isolated from domesticated animals, SFTS patients and ticks was calculated by using the neighbor-joining method with MEGA 5 (*8*). Sequences are labeled with the order of GenBank accession number/name of viral strain/year of isolation. Black circles indicate the original sequences of SFTSV strains obtained from domesticated animals in this study; black triangles indicated the original sequences of SFTSV strains obtained from SFTS patients in 2011 in this study; blank triangles indicate the previously published sequences of SFTSV strains obtained from 11 SFTS patients in 2010 (*1*); and open diamonds indicate the previously published sequences of SFTSV strains obtained from *H. longicornis* ticks in 2010 (*3*).

Virus isolation was attempted on all viral RNA–positive serum samples (n = 103), but isolates were successfully obtained from only 1 sheep (Laizhou), 1 cattle (Laizhou), and 1 dog (Penglai), named SDLZSheep01/2011, SDLZCattle05/2011, and SDPLDog01/2011, respectively. In addition, 10 viral isolates were obtained from SFTS patient serum samples collected in 2011, 9 were from Laizhou (SDLZP01–09/2011) and 1 was from Penglai (SDPL01/2011). Phylogenetic analysis of the S segment of these SFTSV isolates indicated that the viral isolates from sheep, cattle, and dog are genetically close to the 10 SFTS patient-derived isolates in 2011 from the study areas and also to the previous published sequences of viral isolates from 11 SFTS patients reported in 2010 in 6 provinces and *H. longicornis* obtained in 2010 in Laizhou (Figure 6). Pairwise distances analysis showed that all sequences of the isolates from domesticated animals, human patients, and *H. longicornis* shared more than 95% identity, which demonstrated a close evolutionary relationship among those SFTSV isolates from domesticated animals, ticks, and SFTS patients.

## Discussion

In our study of >3,000 serum samples collected from 5 species of domesticated animals in 2 SFTS-endemic counties of Shandong Province, we provided evidence of natural infection and circulation of SFTSV in these animals. We found especially high (>60%) seropositivity of SFTSV-specific antibodies in sampled sheep and cattle, while almost one-half of chickens and one third of dogs tested were seropositive. We detected SFTSV RNA in all 5 species of domesticated animals at a level ranging from 1.7% to 5.3%. A cohort study with 38 sheep and a follow-up study of an infected dog provided insights into the kinetics of natural SFTSV infections in these species. Moreover, the isolation of SFTSV from a sheep, cattle, and a dog provided evidence of SFTSV viremia in domesticated animals.

A previous study in Yiyuan County, Shandong Province, showed a high seroprevalence in goats (83%) (*4*), and another serosurvey of domesticated animals conducted in Jiangsu Province found SFTSV antibody– positive rates of 57% in goats, 32% in cattle, 6% in dogs, 5% in pigs, and 1% in chickens but no antibodies in geese and mice (*5*). Our investigation, conducted in 2 other SFTS-endemic regions in China (Technical Appendix Figure 1), demonstrates a high seroprevalence of SFTSV in sheep (69.5%), cattle (60.4%), dogs (37.9%), and chickens (47.4%), but low rates in pigs (3.1%). Results of seroprevalence of SFTSV infection were consistent and especially high in goats and sheep but low in pigs in these studies. However, our data showed a higher seropositivity of SFTSV-specific antibodies in cattle, dogs, and chickens than the previous report in Jiangsu Province.

In addition, we also found that the SFTSV RNA was detected in all sampled animal species, but the prevalence was low, ranging from 1.7% to 5.3%, which indicates the potential viremia in these animals. The differences in the rates of SFTSV infections among various animal species and regions were statistically significant, perhaps because of varying degrees of exposure to virus-infected ticks or differences in host susceptibility to SFTSV infection. The sheep and cattle in our study regions were grazed on pastures or hills during the day and kept in household backyards at night; the dogs and chickens roamed freely in fields. Pigs were the only animals that did not roam freely. We found that the animals in this study, particularly the sheep and cattle, were heavily infested with ticks. *H. longicornis*, which has been implicated as a vector of SFTSV, is the dominant tick species in these areas. SFTSV RNA had been detected by using a method of real-time RT-PCR from *H. longicornis* ticks collected from grass, cattle, and sheep with a detection rate of ≈2% in disease-endemic areas in previous studies (*3**,**9*), and a viral isolate was obtained from *H. longicornis* ticks on sheep from the same study area of Laizhou in 2010 (*3*). Phylogenetic analysis of the virus isolates obtained from the sheep, cattle, and dog in this study and from *H. longicornis* ticks in our previous study (*3*) showed >95% homology with SFTSV isolates obtained from patients from the same region, which suggests a potential link of SFTSV infections among humans, domesticated animals, and ticks.

Infective virus, a competent insect vector, and susceptible vertebrate hosts must coexist to establish and maintain arbovirus transmission cycles (*10*). Threshold viremia levels of 10^2.0–4.7^ 50% lethal dose/mL have been reported as sufficient for the infection of ticks with Colorado tick fever virus, Russian spring-summer encephalitis virus, and louping ill virus (*11**–**14*). In our study, we detected viral RNA in all domesticated animal species examined, although the viral RNA copy numbers were low (<10^5^ copies/mL). The individual dog in which the clinical course of infection was followed showed a course of acute infection typical of a bunyavirus infection, including the stages of viremia and antibody response. Successful isolation of SFTSV from a sheep, cattle, and a dog confirms the occurrence of SFTSV viremia in these domesticated animals. However, only 3 isolates were obtained from 103 SFTSV RNA–positive serum samples, which may indicate that most infected domesticated animals might have either a low level of viremia, a short period of viremia, or that few infectious virions might be present under the condition of high level of serum antibodies.

During the cohort study of 38 sheep, seroconversion was observed, indicating ongoing circulation of SFTSV in the endemic areas. In 17 of 38 sheep, neutralizing antibodies were observed in the viral RNA in the blood. Although more evidence is needed to illustrate the underlying mechanisms, this case is not the only case of *Bunyaviradae* virus infection. A persistent infection of hantavirus can be established in the rodent reservoir, lasting several months or years with high titers of neutralizing antibodies in the serum (*15**,**16*). Persistent infection of SFTSV in animals has not been shown to date, and a more sensitive nucleotide acid detection method may help to define whether SFTSV infection is prolonged in domestic animals. However, the evidence suggests that SFTSV is circulating among animals and between animals and humans in the endemic areas.

The results of this study are subject to several limitations. First, only 3 viral isolates (1 from each animal species) were obtained and sequenced, which limits the ability to generalize the representativeness of SFTS viral diversity in these areas. Second, SFTSV infections in ticks on animals were not systematically studied to evaluate their relation to SFTSV-infected status or with seroconversion of studied animals, which makes it difficult to associate the infection of SFTSV in ticks directly with that in animals and human patients. Third, we cannot determine whether the virus is pathogenic to the domesticated animals in this study because no adequate examinations have been taken place.

In conclusion, we have provided evidence to show that SFTSV is circulating among several species of domesticated animals and between animals and humans in disease-endemic areas of China. The domesticated animals with high infection rates of SFTSV may act as amplifying hosts for the virus during the epidemic season. More studies are needed to elucidate the SFTSV transmission model in nature, which includes determining the duration of viremia levels and the possibility of persistent infection in each animal species, potential reservoirs, and links of SFTSV infections among humans and domesticated animals. These findings will help formulate effective measures for containing the infection of this emerging pathogen.

Technical Appendix Location of Laizhou and Penglai counties in Shandong Province, China, and serum severe fever with thrombocytopenia syndrome virus RNA copies and antibody detection in domesticated animals from Laizhou and Penglai counties, China, 2011.
