# Supplementary material for: Severe Fever with Thrombocytopenia Syndrome Virus among Domesticated Animals, China
Source: Emerg Infect Dis. 2013 May;19(5):756–63. doi: 10.3201/eid1905.120245 (PMC3647489; doi:10.3201/eid1905.120245)
Supplement: Technical Appendix — Location of Laizhou and Penglai counties in Shandong Province, China, and serum severe fever with thrombocytopenia syndrome virus RNA copies and antibody detection in domesticated animals from Laizhou and Penglai counties, China, 2011. [file 12-0245-Techapp-s1.pdf]

Article DOI: http://dx.doi.org/10.3201/eid1905.120245

Severe Fever with Thrombocytopenia Syndrome Virus among Domesticated Animals, China

Technical Appendix

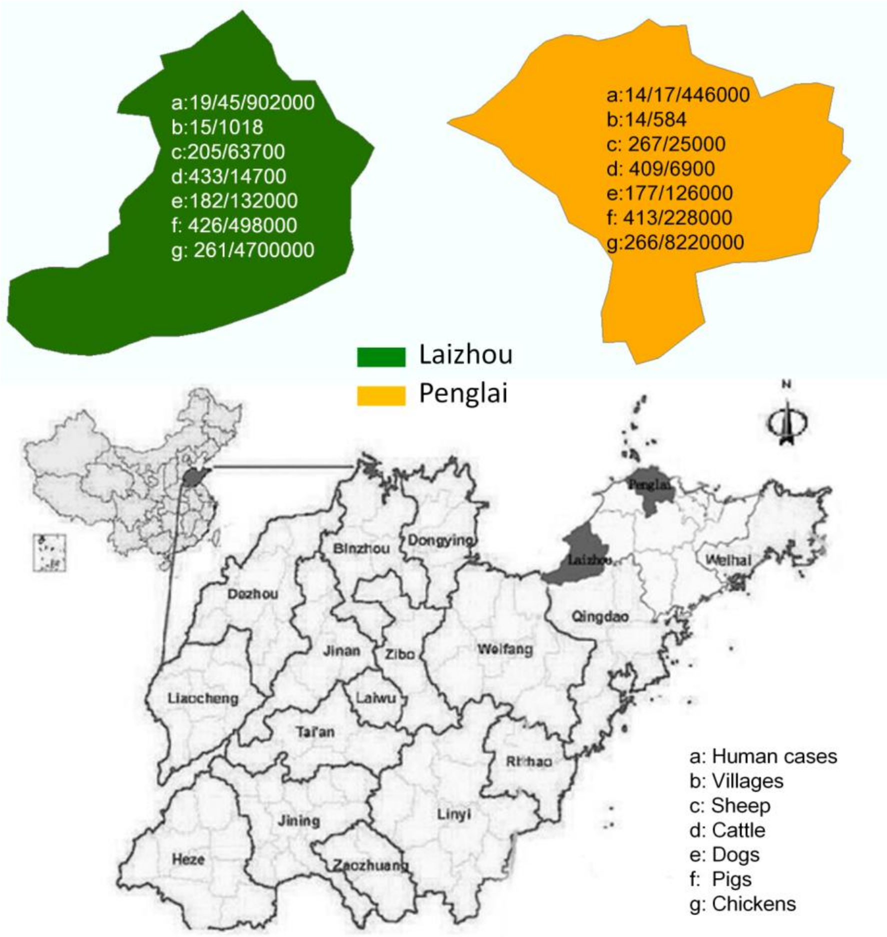

Technical Appendix Figure 1. Location of Laizhou and Penglai counties in Shandong Province, China.

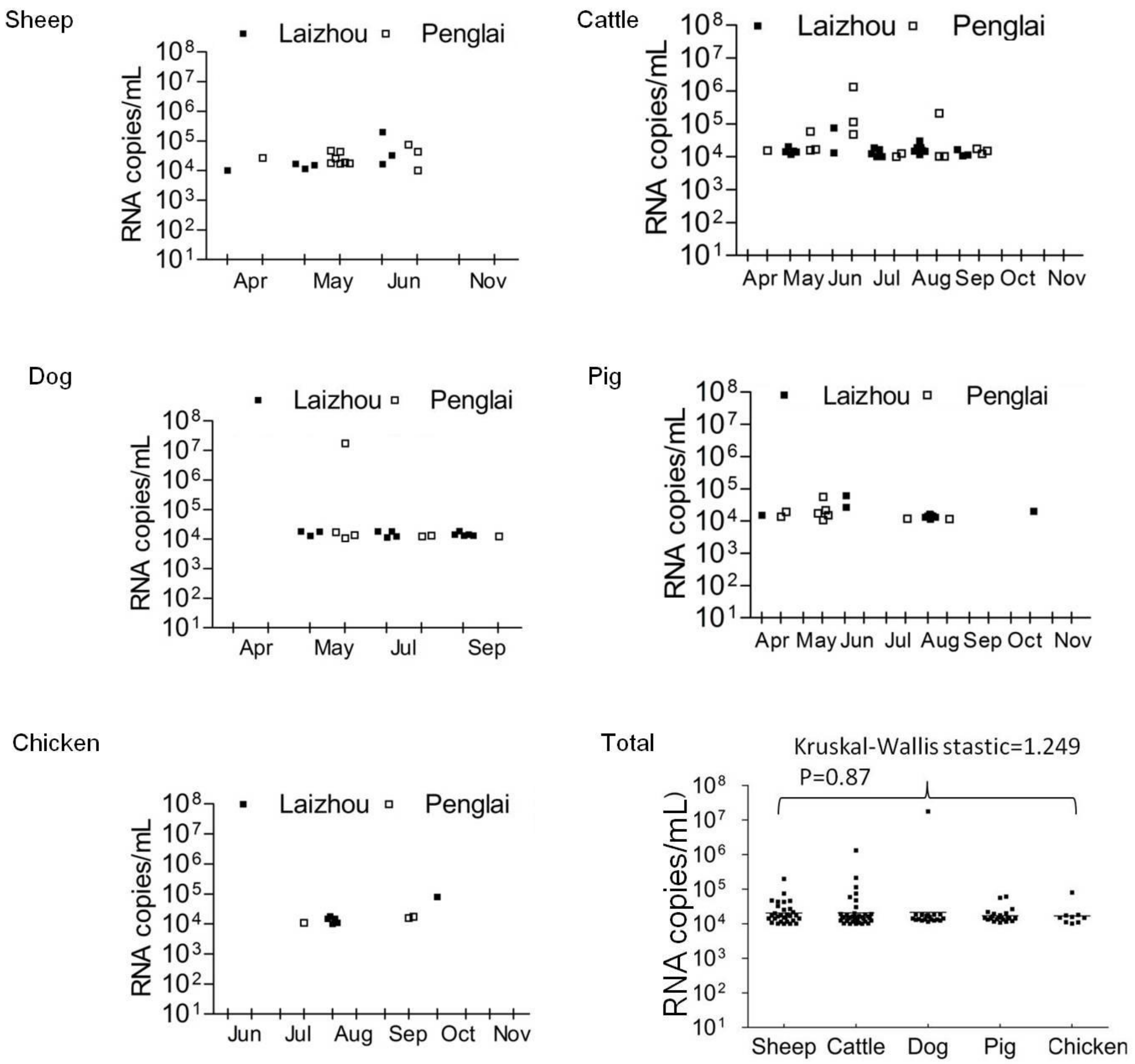

Technical Appendix Figure 2. Serum severe fever with thrombocytopenia syndrome virus RNA copies in domesticated animals from Laizhou and Penglai counties, China, 2011.

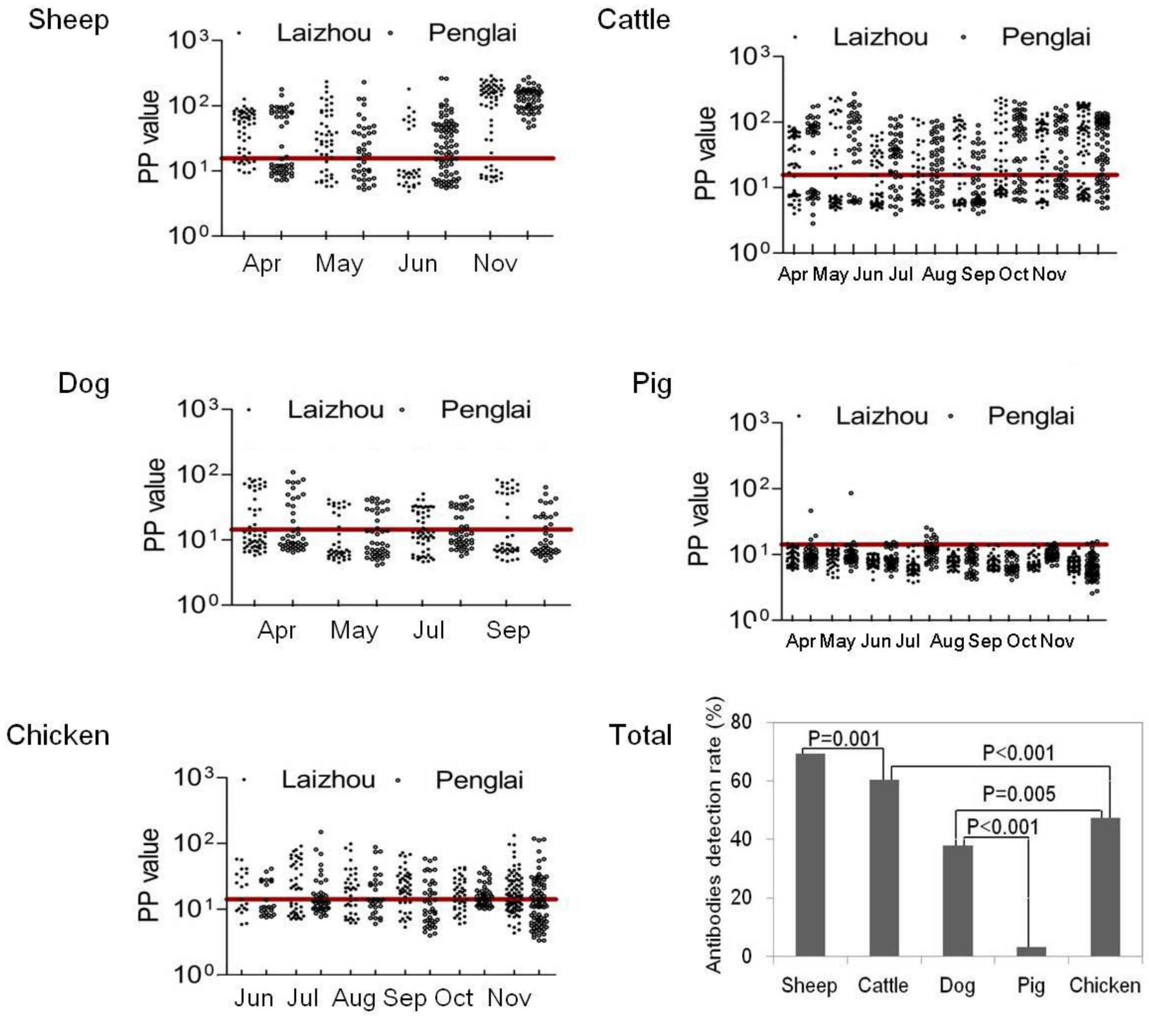

Technical Appendix Figure 3. Serum antibody detection in domesticated animals from Laizhou and Penglai counties, China, 2011.
